# Survey of Potential Drug Interactions, Use of Non-Medical Health Products, and Immunization Status among Patients Receiving Targeted Therapies

**DOI:** 10.3390/ph17070942

**Published:** 2024-07-14

**Authors:** Réka Rajj, Nóra Schaadt, Katalin Bezsila, Orsolya Balázs, Marcell B. Jancsó, Milán Auer, Dániel B. Kiss, András Fittler, Anna Somogyi-Végh, István G. Télessy, Lajos Botz, Róbert Gy. Vida

**Affiliations:** 1 Department of Pharmaceutics, Faculty of Pharmacy, University of Pécs, 7624 Pécs, Hungaryfittler.andras@pte.hu (A.F.);; 2 Central Clinical Pharmacy, Clinical Center, University of Pécs, 7624 Pécs, Hungary

**Keywords:** targeted therapy, biological therapy, chronic immunological disorders, clinical pharmacy service, dietary supplements, drug interactions, patient interview, rheumatoid arthritis, vaccines

## Abstract

In recent years, several changes have occurred in the management of chronic immunological conditions with the emerging use of targeted therapies. This two-phase cross-sectional study was conducted through structured in-person interviews in 2018–2019 and 2022. Additional data sources included ambulatory medical records and the itemized reimbursement reporting interface of the National Health Insurance Fund. Drug interactions were analyzed using the UpToDate Lexicomp, Medscape drug interaction checker, and Drugs.com databases. The chi-square test was used, and odds ratios (ORs) were calculated. In total, 185 patients participated. In 53% of patients (n = 53), a serious drug–drug interaction (DDI) was identified (mean number: 1.07 ± 1.43, 0–7), whereas this value was 38% (n = 38) for potential drug–supplement interactions (mean number: 0.58 ± 0.85, 0–3) and 47% (n = 47) for potential targeted drug interactions (0.72 ± 0.97, 0–5) in 2018. In 2022, 78% of patients (n = 66) were identified as having a serious DDI (mean number: 2.27 ± 2.69, 0–19), 66% (n = 56) had a potential drug–supplement interaction (mean number: 2.33 ± 2.69, 0–13), and 79% (n = 67) had a potential targeted drug interactions (1.35 ± 1.04, 0–5). Older age (>60 years; OR: 2.062), female sex (OR: 3.387), and polypharmacy (OR: 5.276) were identified as the main risk factors. Screening methods and drug interaction databases do not keep pace with the emergence of new therapeutics.

## 1. Introduction

Drug-related problems (DRPs) pose significant challenges in clinical practice. In the last two decades, several approaches have been developed to ensure patient safety because of the clinical and economic impact of DRPs and DRP-induced adverse drug reactions (ADRs) [1,2,3,4].

Pharmaceutical Care Network Europe (PCNE) has developed a generally accepted and widely used definition of a drug-related problem as a DRP, defined as an event or circumstance related to drug therapy that actually or potentially interferes with desired health outcomes [5]. Potential drug–drug interactions (pDDIs) are a type of preventable DRP, and their screening and prevention can improve patient safety in any healthcare setting [6,7]. Although numerous studies have been published on the investigated drug–drug interactions, the reported prevalence varies widely. At the same time, the healthcare setting, population, available screening methods, and therapeutic regimens may differ. Different types of categorizations related to drug–drug interactions (DDIs), clinically significant DDIs, and other types of DDIs add to the complexity of drug–drug interactions [8,9,10]. DDIs can have several consequences, the most common of which are adverse effects on morbidity, mortality, length of hospital stay, healthcare costs, and quality of life [8,11]. Although it is difficult to assess the role of DDIs alone in these outcomes, and the number of clinical trials focusing on drug–drug interactions is considered to be low, published data show that 17–27% of complications in hospitalized patients are due to DDIs [12,13]. Common risk factors include age, sex, changes in pharmacokinetic parameters, polypharmacy, medication errors, and comorbid conditions [14,15]. In a 2014 review, Dechanont et al. reported a median prevalence of 1.1% (367 DDI cases/47,976 DDI patients) among hospital admissions [16]. In oncology, it is estimated that 2% of hospital admissions are due to ADRs caused by DDIs [17,18]. However, a recent Serbian study reported that 9.69% of hospital admissions were due to DDIs [18]. A real-world study of 1799 participants in a multivariate model also revealed an association between DDIs and hospital admission (OR = 1.29, 95% CI 1.04–1.58, *p* = 0.018) [19].

Drug interactions are generally defined as the concomitant use of other drugs or substances that affect the effects of a drug. However, not only other substances but also the general condition or disease can change the effect of drug therapy (drug–disease interaction) by changing the pharmacokinetics of a medicines, like liver or kidney disease. When we categorize drug interactions, the counteracting partner may be another drug (drug–drug interaction), food/beverages, dietary supplements, other complementary and alternative (CAM) therapies (drug–nutrient/food/CAM interaction), formulation excipients (in the case of incompatibilities/drug–drug interactions), and environmental factors (e.g., smoking) [20,21,22,23].

Although most publications highlight the potential of DDIs to cause ADRs, some pharmacokinetic and pharmacodynamic DDIs are beneficial and used as a combination in clinical practice, such as reversal of opioid-induced respiratory depression, and combinations used in anesthesia, cardiology, and rheumatology (e.g., methotrexate combined with folic acid) [24,25,26].

A survey conducted in 2012 at our institution revealed that both inpatients and outpatients took supplements (85.5% of 200 patients, mean number 2.5) that were not registered in the medical records. Women were more likely to take supplements, and there was a significant risk of potential drug interactions. The general conclusions of the study underscore the necessity of consulting multiple interaction databases to identify all relevant interactions and emphasize the importance of incorporating supplement use into medication histories [27]. Another study among psychiatric patients in 2017 showed similar results; the average number of supplement products taken by patients was 4.7, and 90.8% of patients took at least one supplement [28]. Other observations that brought our research to life include complications and unresolved issues in the pDDI screening practice. With the emerging use of dietary supplements and novel therapeutics, gathering information about these in real life is crucial. In our study, we aimed to identify pDDIs in patients receiving targeted therapies and to assess whether online DDI screening tools are suitable for preventing drug–drug and drug–supplement interactions, as previous studies have shown that there are significant discrepancies among databases, and integration of interaction alerts involving supplements remains limited [27,29,30,31,32].

The itemized reimbursement system represents a unique case-by-case reimbursement approach for high-priced innovative products in Hungary. Drugs financed by this technique are available only at designated clinical centers, providing hospital pharmacy staff with deeper insight into these therapies as well as regular contact with the patients involved [33,34].

## 2. Results

### 2.1. Medication Therapy and Drug Interactions in 2018/2019

In the 2018–2019 survey, out of 100 patients, 57 (57%) were female, and 43 (43%) were male. The majority of patients belonged to the 50–59 age group (n = 33, 33%). Sixty-nine (69%) patients were from the rheumatology and immunology departments, and 31 (31%) were from the dermatology clinic. The primary diagnoses were rheumatoid arthritis (n = 39, 39%), plaque psoriasis (n = 28, 28%), ankylosing spondylitis (n = 21, 21%), and other conditions (n = 12, 12%; see Table 1). The mean disease activity indices were as follows: 3.08 (±1.57) for Disease Activity Score 28 (DAS28), 14.07 (±18.71) for Bath Ankylosing Spondylitis Disease Activity Index (BASDAI), 1.49 (±3.4) for Psoriasis Area and Severity Index (PASI), and 1.31 (±4.54) for Dermatology Life Quality Index (DLQI). The mean time from diagnosis of the primary disease requiring targeted therapy was 14.89 (±9.29; 0.7–49) years. Patients had been receiving targeted therapy for an average of 5.73 (±4.32; 0.3–20) years. The rate of therapeutic change involving targeted therapy was 40% (n = 40/100), with 11 patients (11%) undergoing changes more than once (ranging from two to four times). An assessment of efficacy from individual medical records revealed that 31% (n = 31) of the patients achieved complete remission, while 43% (n = 43) achieved partial remission. Moreover, 32% (n = 32) of the patients received adalimumab (Humira, sc), 16% (n = 16) received infliximab (Remicade, iv), and 15% (n = 15) received tocilizumab (RoActemra, sc). Other therapies included etanercept (Enbrel, sc, n = 11, 11%), rituximab (MabThera, iv, n = 8, 8%), certolizumab pegol (Cimzia, sc, n = 5, 5%), golimumab (Simponi, sc, n = 5, 5%), ustekinumab (Stelara, iv, n = 4, 4%), intravenous immunoglobulin (Privigen, iv, n = 2, 2%), and, in one patient, abatacept (Orencia, iv, n = 1, 1%) and secukinumab (Cosentyx, sc, n = 1, 1%).

In the 2018–2019 population, the mean number of prescribed medications was 6.02 (±4.28; 0–22); for OTC medications, it was 1.4 (±1.35; 0–5); and for non-medical health products/supplements, it was 0.52 (±1.04; 0–6). A total of 66% (n = 66) of patients used over-the-counter medications, and 30% (n = 30) used at least one non-medical health product or supplement in the two weeks preceding the interview. Serious potential DDIs were identified in 53% (n = 53) of the patients (mean number: 1.07 ± 1.43; 0–7), potential drug–supplement interactions in 38% (n = 38) (mean number: 0.58 ± 0.85; 0–3), and potential targeted drug interactions in 47% (n = 47%) (0.72 ± 0.97; 0–5). More than five drugs were prescribed to 57% (n = 57) of the patients. We used three different DDI screening programs and identified 573 alerts in the UpToDate Lexicomp, 585 in the Drugs.com, and 552 in the Medscape drug interaction checker. Of these, 15 were potentially serious pDDIs in the UpToDate Lexicomp database, 91 in the Drugs.com database, and 53 in the Medscape drug interaction checker. For drug–supplement interactions, we found 58 (UpToDate Lexicomp database), 20 (Drugs.com database), and 20 (Medscape’s drug Interaction Checker). Lastly, for targeted interactions, 30 were from UpToDate Lexicomp, 68 were from Drugs.com, and 28 were from Medscape.

### 2.2. Medication Therapy and Drug Interactions in 2022

In 2022, among the 85 patients, 64% (n = 54) were female, and 36% (n = 31) were male. Most patients were in the 60–69 age group (n = 27, 31.8%). All patients were recruited from the Department of Rheumatology and Immunology. The most common diagnoses were rheumatoid arthritis (n = 48, 56.5%), ankylosing spondylitis (n = 17, 20.0%), and psoriatic arthritis (n = 14, 16.5%). The mean disease activity index was 2.98 (±1.18) for DAS28 and 6.81 (±7.10) for BASDAI.

Based on the 2022 data, the mean time from diagnosis of the primary disease requiring targeted therapy was 14.79 (±9.14; 0–43) years. Patients had been receiving targeted therapy for an average of 6.95 (±4.39; 0.2–17) years. The rate of therapeutic change during the years of targeted therapy was 65.8% (n = 56), with 37 (43.6%) patients switching therapies multiple times (2–8 times). Based on the assessment of individual medical records, 16% (n = 14) of the patients were in complete remission, whereas 55% (n = 47) were in partial remission. Of the patients, 27.1% (n = 23) received adalimumab (Hyrimoz, sc), 22.4% (n = 19) received tocilizumab (RoActemra, sc), and 14.1% (n = 12) received etanercept (Enbrel, sc). Other targeted therapies included golimumab (Simponi, sc, n = 8, 9.4%), infliximab (Zessly, iv, n = 7, 8.2%), tofacitinib (Xeljanz, oral, n = 4, 4.7%), certolizumab pegol (Cimzia, sc, n = 3, 3.5%), baricitinib (Olumiant, oral, n = 3, 3.5%), abatacept (Orencia, iv, n = 2, 2.4%), secukinumab (Cosentyx, sc, n = 2, 2.4%), upadacitinib (Rinvoq, oral, n = 1, 1.9%), and ixekizumab (Taltz, sc, n = 1, 1.9%).

In 2022, the average number of prescribed drugs was 6.86 (±4.89; 1–23); for OTC drugs, it was 0.99 (±0.96; 0–4); and for non-medical health products/supplements, it was 2.41 (±1.42; 0–7). OTC drugs were used by 66% of the patients (n = 56), and 88% (n = 75) used at least one non-medical health product or supplement. Severe DDIs were identified in 78% (n = 66) of patients (mean number: 2.27 ± 2.69; 0–19), potential drug–supplement interactions in 66% (n = 56) (mean number: 2.33 ± 2.61; 0–13), and potential targeted interactions in 79% (n = 67) (1.35 ± 1.04; 0–5). More than five drugs were prescribed to 81% of patients (n = 69). We identified 601 drug interaction alerts in the UpToDate Lexicomp, 1213 in the Drugs.com, and 888 in the Medscape drug interaction checker. From these, 119 had severe pDDIs in UpToDate Lexicon, 161 in Drugs.com, and 85 in Medscape. For drug–supplement interactions, 95 (UpToDate Lexicomp database), 106 (Drugs.com database), and 172 (Medscape’s drug interaction checker) were identified. For targeted drug interactions, 38 interactions were found in the UpToDate Lexicomp database, 114 in the Drugs.com database, and 43 in the Medscape drug interaction checker. The main characteristics of the survey population during the two periods are presented in Table 1.

Examples of the categories of serious pDDIs identified in the survey are presented in the Supplementary File (Tables S1–S6). In general, the main pDDIs were the same in both study periods. However, the introduction of Janus kinase (JAK) inhibitors into the therapeutic armamentarium has increased the risk of severe pDDIs via the CYP3A4 pathway. Thus, JAK inhibitors should be avoided with strong CYP3A4 inducers and grapefruit juice. The risk of cardiovascular toxicity should also be mentioned, although the DDI screening programs did not show any results when screening with NSAIDs and coxibs [35].

In the case of targeted therapies, the main pDDIs identified in online databases are in line with the Summary of Product Characteristics, with interactions between adalimumab or other TNF-α inhibitor monoclonal antibodies and alprazolam, atorvastatin, and amlodipine being the most frequently noted examples.

Two interesting pDDIs not found in the databases are highlighted for 2022: the interaction between duloxetine–gabapentin and adalimumab or other TNF-α inhibitors and etanercept–semaglutide (Table S4) [36,37]. There are some theoretical possibilities for this interaction, and in the case of duloxetine, a case report can be found in the scientific literature (see Discussion). We wanted to communicate these potential interactions.

**Figure 1:**
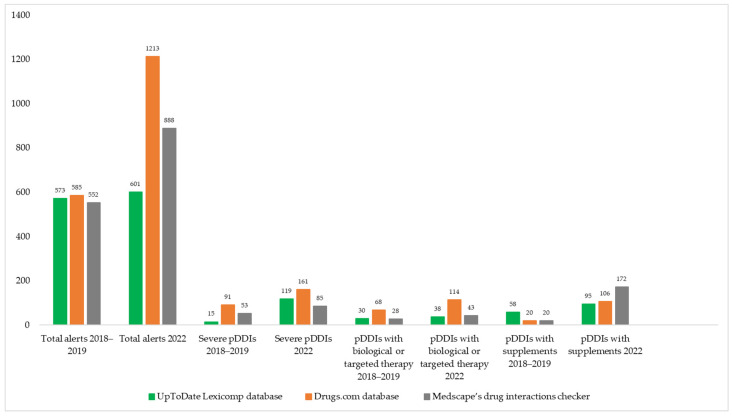
Number of pDDIs and comparison of the three DDI screening databases in 2018–2019 and 2022 years.

The prevalence of different types of potential drug interactions is shown in Figure 1.

### 2.3. Supplement Use

In 2018–2019, the top three non-medical health products/supplements were vitamin D (n = 33, 33%), vitamin D and calcium combination (n = 17, 17%), and vitamin C (n = 17, 17%). Although only one or two patients took plants or herbal supplements such as milk thistle (n = 2, 2% in 2018/2019 and n = 3, 3.6% in 2022), ginger (n = 1, 1.2% in 2022), *Ginkgo biloba* (1 patient, 1% in 2018), turmeric (1 patient in each year, 1% in 2018/2019 and 1.2% in 2022), green tea (1 patient in each year, 1% in 2018/2019 and 1.2% in 2022), and St. John’s wort (1 patient in 2018/2019), it should be mentioned that their potential to interfere with drug therapy is relatively high.

The main motivations named by participants in 2018–2019 for using non-medical health products or supplements were to protect the liver, prevent infections, and reduce anxiety or sleep disorders.

In 2022, the top three non-medical health products and supplements were vitamin D (n = 61, 71.8%), vitamin C (n = 38, 44.7%), and calcium (n = 25, 29.4%). Although there was a significant decrease in the use of phytotherapy, with one or two patients taking plants or herbal supplements such as milk thistle, ginger, turmeric, green tea, and echinacea, their potential to interfere with drug therapy requires attention.

The main motivations for using non-medical health products or supplements in 2022 were reducing adverse drug reactions, preventing COVID-19 infection, and assisting in healing.

Thirty-three patients (33%) experienced self-reported adverse effects in 2018–2019, including nausea (n = 3, 9.1%), urinary tract infection (n = 3, 9.1%), hypertension (n = 3, 9.1%), fatigue (n = 3, 9.1%), hair loss (n = 2, 6.1%), and dermatological problems (n = 2, 6.1%). In one patient (3.0%), severe infection caused temporary interruption of targeted therapy. In 2022, 21 patients (24.7%) experienced self-reported AEs, including dermatological problems (n = 3, 14.3%), prostate problems (n = 1, 4.8%), diarrhea (n = 3, 14.3%), and non-serious upper respiratory tract infections (n = 3, 14.3%).

Table 2 shows the non-medical health products and supplements used by our study population in both years.

As of 2022, vitamin D has become the most common supplement; the most critical potential supplement–drug interactions are related to this vitamin. In 2018–2019, we identified one patient taking St. John’s wort, whereas in both years, potential interactions were identified with omega-3 fatty acids and milk thistle.

### 2.4. Immunization Status

**Figure 2:**
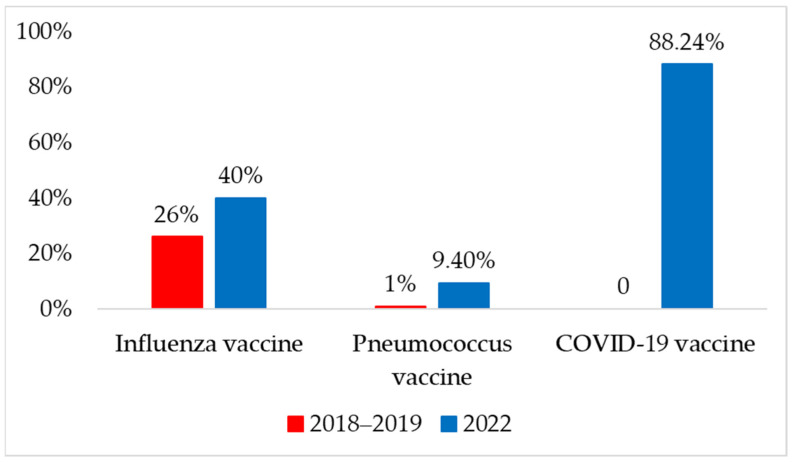
Self-reported immunization status in 2018–2019 and 2022 years.

When we assessed the vaccination status of the patients in 2018–2019, we found a 26% (n = 26) self-reported vaccination rate for influenza and 1% (n = 1) for pneumococcal vaccine. Other optional vaccines, such as adult BCG, tick-borne encephalitis, and tetanus, were also identified in one-third of the patients (n = 33, 3%). When the survey was repeated in 2022, self-reported vaccination rates increased to 40% for influenza (n = 34) and 9.4% for pneumococcus (n = 8). The COVID-19 vaccination rate was the highest at 88.2%. In 2022, no other optional vaccines were identified in the survey population in the previous year. A comparison of vaccination rates between the two years is shown in Figure 2.

### 2.5. Statistical Analysis

The odds ratios (OR), 95% confidence intervals, and *p*-values for the independent variables that demonstrated a significant association with pDDIs (*p* < 0.05) are presented in Table 3. We found a statistically significant association and therefore a higher risk of severe pDDI with age (>60 years; OR = 1.901, 95% CI = 1.022–3.537, *p* = 0.041), female sex (OR = 3.129, 95% CI 1.674–5.849, *p* < 0.001), and polypharmacy (≥5 drugs) (OR = 10.50, 95% CI 5.122–21.532, *p* < 0.001). For supplement interactions, age (>60 years; OR = 3.173, 95% CI = 1.732–5.813, *p* < 0.001), sex (female; OR = 2.651, 95% CI = 11.444–4.865, *p* < 0. 001), polypharmacy (≥5 drugs) (OR = 11.518, 95% CI = 5.167–25.674, *p* < 0.001), and use of dietary supplements (OR = 5.175, 95% CI = 2.746–9.752, *p* < 0.001) increased the risk. Age (>60 years; OR: 2.062), female sex (OR: 3.387), and polypharmacy (OR: 5.276) were identified as risk factors for potential biological or targeted drug interactions.

We carried out another analysis with just the rheumatology and immunology patients (n = 154) and found that in case of severe pDDI and targeted pDDI, the age (>60 years) was no longer significant risk factor (shown in Table 4).

## 3. Discussion

In our study, we observed a high prevalence of pDDIs, as in 2018–2019, 53% of the patients were identified as exposed to at least one serious DDI, whereas in 2022, this ratio was 78%. A similar increase was observed for potential drug–supplement and targeted drug interactions. When examining the risk factors, we identified older age (>60 years) and polypharmacy (≥5 drugs) for each pDDI category (severe, supplement, and targeted therapy) and female sex as the main risk factors not mentioned in previous publications [38,39,40]. In case of the rheumatology and immunology subgroup (n = 154) we found that age (>60 years) was no longer a risk factor for severe and targeted pDDI. The possible explanation for this is the lower ratio of patients over 60 years in the dermatology group (n = 31).

A mixed-methods (cross-sectional and cohort) study in Denmark used hospital electronic health records from 2008 to 2016 to identify potential drug–drug interactions [41]. The study included all inpatients who received two or more medications during their admission to measure the prevalence of pDDIs and their associations with adverse outcomes. Of the 2,886,227 hospital admissions (945,475 patients; median age, 62 years; 54% female; median number of drugs, 7), 1,836,170 admissions (63.61%), including 659,525 patients (69.75%), were exposed to at least one pDDI. Meropenem–valproic acid, domperidone–fluconazole, imipramine–terbinafine, agomelatine–ciprofloxacin, clarithromycin–quetiapine, and piroxicam–warfarin pDDIs were associated with increased mortality [41].

A Brazilian cohort study of 103 patients with rheumatology, published in 2011, reported a polypharmacy rate of 95.1% and a mean number of 3.0 ± 1.2 interactions per patient in 74 patients (71.84%). All potential DDIs, including methotrexate and omeprazole (29.3%), diclofenac sodium (17.6%), and metamizole (13.2%), were major interacting pairs. This study used the Micromedex DDI screening database [42]. A more recent retrospective study of 200 patients with RA from 2012 to 2017 in Malaysia found that the most common DRPs according to the PCNE classification (version 5.01) were adverse reactions (38.8%), followed by drug interactions (33.6%) and drug choice problems (14.5%). The factors associated with DRPs were polypharmacy, multiple comorbidities, hyperlipidemia, osteoarthritis, and renal impairment. The most common interactions were detected with DMARDs such as methotrexate and sulfasalazine, with concomitant use of prednisolone, simvastatin, amlodipine, and omeprazole [43].

Another multicenter, cross-sectional study from Brazil evaluated 792 patients (89% female, median age 56.6 years). The main results are comparable with our findings, as the population was similar (median disease duration was 12.7 years, and median Disease Activity Score 28 was 3.5; patients with mild activity), with therapeutic regimens of corticosteroids (47%), nonsteroidal anti-inflammatory drugs (9.1%), synthetic disease-modifying anti-rheumatic drugs (90.9%), and biologic disease-modifying anti-rheumatic drugs (35.7%) [43]. However, our study included patients receiving targeted therapy; the polypharmacy rate was high at 67.9% in this study [44] compared with our study (57% in 2018–2019 and 81.2% in 2022.

A retrospective cohort study specific to Central European patients with RA from 2017 to 2019, with a systematic literature review including the results of 175 patients, reported that the average number of medications per patient was 6.6 ± 3.5, while the prevalence of polypharmacy was 33.7%. As mentioned above, our results were higher on average (61.6%) based on the literature review of 24,446 patients in this publication [45].

A prospective observational cohort study published in 2019 based on registry data from the British Society for Rheumatology Biologics Register (BSRBR-RA) comparing the initiation of biologic therapy with DMARDs reported an analysis of 22,005 patients, 83% of whom were on biologic therapies and had a mean age of 57 years, median disease duration of 12 years, and 6.15 (±1.23) baseline DAS-28 in the study population. Polypharmacy was 35.9%. This research has provided valuable points to consider when optimizing the medication therapy of patients with rheumatology on biologic therapy. Bechman et al. concluded that polypharmacy could be used as a predictor of clinical outcomes: For each additional medication a patient is taking after his/her DMARD therapy, they are 8% less likely to achieve a good treatment response when starting a biologic therapy, and there is an increased risk (13%) of developing serious adverse events (SAEs) [46].

There are many studies in different conditions from many countries, and the comparability of these studies and their results is relatively low. Therefore, the alterations in the prevalence of pDDIs may be due to different DDI databases, prescribing practices, and study populations [40]. However, the risk factors identified and published in these studies may help increase awareness and motivate targeted prevention and management strategies. Older patients, multiple comorbidities, new prescriptions (polypharmacy), chronic kidney disease (CKD), and patients visiting more physicians are at higher risk of developing pDDIs, which is supported by our findings, too [47,48,49,50,51].

Although biological medicines entered the market more than 20 years ago, limited data are available regarding their drug–drug interactions. Early publications highlighted the interaction of tocilizumab with simvastatin, with a possible mechanism of altered IL-6 levels affecting the CYP450 enzyme system (predominantly CYP3A4), and the potential effect of immunomodulators such as methotrexate on the disposition of anti-TNF-α biologics (e.g., infliximab). These pDDIs were also identified in our study. Based on our observations as well, it should be emphasized that close monitoring should be initiated whenever targeted therapy is started, especially if the patient is taking another drug with a narrow therapeutic index (e.g., anticoagulant) [36,52,53]. Further, recent studies have suggested the potential role of cytokines such as IL-8, IL-10, IL-17, IL-1β, IL-2, tumor necrosis factor (TNF)-α, and interferon (IFN)-γ, which may also suppress CYP450 enzymes [54].

A review of drug–drug interactions for biologics used to treat psoriasis published in 2020/21 found that, in general, there are few case reports on the topic and scarce studies with small sample sizes, resulting in a low level of evidence [55]. More studies and the collection of data from real-world studies may help increase the evidence level and develop specific guidelines for patients receiving targeted therapies. Our results are consistent with this review, as most pDDIs combined different immunosuppressive therapies used with therapeutic intent in patients with rheumatology and psoriasis. We also identified patients with potential interactions via the CYP450 enzyme system (e.g., TNF-α blockers, statins, TNF-α blockers, and alprazolam). Armanious and Vender cited a case study in which the interaction between adalimumab and pregabalin/duloxetine was discussed, and the introduction of adalimumab therapy resulted in worsening of the patient’s neuropathic pain [36,55]. Duloxetine interactions were also identified in our survey as well (Supplementary File Table S4).

Further pDDIs resulting in abnormal liver enzyme levels in patients on concomitant etanercept and indomethacin therapy have also been reported in two cases [55,56]. A comprehensive drug–drug interaction search was published by Pflugbeil et al. in 2020, who reported up-to-date information on potential drug interactions for drugs used in the treatment of rheumatoid arthritis and psoriatic arthritis [26]. We identified the same pDDIs for DMARDs and t/bDMARDs in our patient group (see Supplementary File Tables S1–S4).

As highlighted in the introduction, screening for potential drug–drug interactions is complicated due to the heterogeneity of databases; therefore, it is recommended to use more than one database. Relying on the Summary of Product Characteristics alone is not sufficient, as SmPC is a sub-optimal source of information for biological and complementary drug interactions. These DDI screening programs can increase patient safety under the supervision and evaluation of a clinical pharmacist [27,29,30,31,57]. As more research and publications are published, the number of databases for screening drug interactions can be increased. Our experience confirms that post-marketing and real-world data collection should be developed as well as follow-up of pre-clinical and clinical drug interaction studies (with the challenge of long elimination half-lives of monoclonal antibodies) because SmPCs are the primary source of information for drug interaction screening in most pharmacy dispensing computer programs.

Prevention strategies include introducing various clinical pharmacy services in ward or community settings. Dutch studies published some decades ago reported that medication reviews performed by clinical pharmacists effectively reduced pDDIs [58,59].

Despite advances in medication and exercise therapies, the popularity of CAM remains high, and the potential benefits and adverse effects should also be considered by healthcare professionals when treating patients with chronic inflammatory diseases.

In our study, we identified the frequent use of CAM products, like herbs and dietary supplements including chondroitin sulfate, copper, *Echinacea* sp., *Gingko biloba*, glucosamine, green tea, and S-adenosyl-L-methionine (SAMe). A similar observation was published in 2009 based on an institutional registry study of 166 patients with RA in the United States [60].

A Hungarian specialty is Béres drops, with trace elements as the corroborative agent. Béres drops have been an authorized medicine in Hungary since 2000 to treat trace element deficiencies, and they contain various trace elements and minerals in supraphysiological quantities. The SmPC of the product states the following warning for pDDIs: “At least 1 h should pass between taking the preparation and other medicines! In order to avoid excessive intake and antagonistic interactions, the use of other preparations containing trace elements.” [61].

Baig and DiRenzo mentioned other CAM and traditional Chinese medicines (TCM) modalities used by patients in a 2020 publication with little or no evidence of efficacy in patients with RA, such as omega-3 fatty acids, gamma-linolenic acid, probiotics, and thunder god vine (*Tripterygium wilfordii*). The traditional Chinese herb has been associated with common adverse effects such as nausea and liver function abnormalities, and its use for medical purposes in Europe is not recommended [62]. The interactions between Western medicines and TCM are known [63,64,65] but are rarely mentioned in the Western interaction databases.

A scoping review published in 2019 on the complementary use of natural products reported an overall prevalence of 47% worldwide, and the authors found no differences according to geographic region. On average, 47% of patients found these products effective, whereas 13% reported adverse effects. The supplements used included green tea products, marine oils, glucosamine, vinegar, chondroitin, propolis, colostrum, green-lipped mussel extract, methylsulfonylmethane (MSM), and gin-soaked raisins. Other products containing botanical ingredients such as garlic, ginger, ginkgo, aloe vera, St. John’s wort, valerian, turmeric, cat’s claw, evening primrose oil, linseed oil, and *Echinacea* sp. have also been reported [66].

A web-based survey conducted in the United States in 2020, which specifically focused on the use of OTC natural product-based (nonvitamin and nonmineral) dietary supplements (NVNM DS) in patients with RA, found a prevalence of current use of 49.6% (n = 303) for NVNM products, 83.5% (n = 510) for vitamins and minerals, and 87.6% (n = 535) for all dietary supplements in 611 subjects. Among the products identified, turmeric, ginger, and fish oil/ω-3 (n-3) polyunsaturated fatty acids (PUFA) were the top three, but flaxseed, *Boswellia* sp., milk thistle, probiotics, coenzyme Q10, and cannabis-derived products were also mentioned by the patients. The reported products were vitamin D, folate, multivitamins, calcium (the top four as a standard part of pharmaceutical therapy), vitamin B_12_, vitamin C, vitamin B_6_, magnesium, zinc, and vitamin B complex. The authors also highlighted the potential adverse effects of concomitant use of methotrexate and turmeric, which have been associated with hepatotoxicity [67].

Numerous supplements and some theoretical or in vitro data support potential interactions; however, plants with high interaction potential may affect several drugs and the efficacy or safety of a patient’s therapy; therefore, any patient using St. John’s wort, cannabis, green tea, or echinacea should be closely monitored [68].

A suboptimal vaccination rate was found in our study; unfortunately, these results are comparable with another study that assessed the prevalence of influenza and pneumococcal vaccination in patients with chronic obstructive pulmonary disease in Hungary—another population for which vaccination is highly recommended by guidelines. Fekete et al. conducted a retrospective, population-based cohort study of 250 patients with COPD (mean age 66.62 ± 8.3 years, 56.4% female) in 2019 and found an overall prevalence of 23.6% for influenza and 10.8% for pneumococcal vaccination [69].

The numbers in Figure 2 demonstrate a statistically significant increase in the ratio of patients vaccinated with influenza or pneumococcal vaccines, as an indirect effect of coronavirus vaccinations. It is unfortunate that the vaccination uptake tendencies in this region are far different from the numbers observed in Western countries.

A study of the vaccination status of patients with rheumatoid arthritis in the United States in 2013, published by Sandler et al., reported that of 102 recruited patients (85.3% female and mean age 57.8 years), 79.4% had self-reported influenza vaccination, 53.9% had pneumococcal vaccination, and 7.8% had herpes zoster vaccination. There was a significant discrepancy between the vaccination status reported by the patients and the data found in the electronic health records [70]. A retrospective cohort study using an extensive primary care database of adult patients with RA treated with non-biological immunosuppressive therapy from 2000 to 2013 in the U.K. (n = 15,724) reported vaccination rates of 80% for influenza and 50% for pneumococcal vaccination. Older patients, those with more comorbidities, and those with more healthcare visits were more likely to update their immunization status with both vaccines [71]. A Canadian observational study assessed the uptake of herpes zoster (HZ), influenza, and pneumonia vaccines among 98 patients with RA using a self-administered questionnaire in an academic rheumatology clinic between 2018 and 2020. Of the patients, 72.4% had received at least one influenza vaccine in 2017–2019. The rates of herpes zoster and pneumococcal vaccination were 18.4% and 36.7%, respectively. The main barriers to vaccination included personal preferences not to be vaccinated, cost, concerns about interactions with treatment, age under 65 years, and not knowing that they should be vaccinated [72].

A study assessing vaccination status conducted in Germany in 2021 recruited 222 adult outpatients (mean age 62.9 ± 13.9 years) with autoimmune inflammatory rheumatic disease (AIIRD) during regular consultations. Approximately 68.5% of the participants were vaccinated against influenza, 34.7% against *Streptococcus pneumoniae*, and 13.1% against herpes zoster. Patients with previous experience with influenza vaccination, over 60 years of age, female sex, and with the use of glucocorticoids were more likely to receive pneumococcal vaccinations. The authors noted that an increase in the frequency of vaccination and the COVID-19 pandemic may have had a positive effect [73]. Other studies have also reported a lower rate of pneumococcal vaccination [74].

### Strengths and Limitations

Our study is the first to describe biological drug–drug interactions among Hungarian immunological patients. Few studies have been published from a drug–drug interaction perspective, making our research a valuable addition to the real-world knowledge of pDDIs involving targeted therapies. As an increasing number of patients receive original and biosimilar biological therapies, it is important to understand, prevent, and manage the pDDIs of these drugs in combination with other therapies. The results demonstrated above indicate that our study has some limitations. The results should be generalized or applied cautiously as they were conducted in a specific patient population and at a specific level of care (outpatient clinic) in Hungary in a limited number (n = 185) and even smaller when forming two groups. As a result, not all findings are generalizable to patient populations in other countries with different prescribing practices and patient characteristics. Even though the authors should highlight that the targeted therapies used in Hungary are the same as those used in other countries, the only difference is how many patients can access these therapies and when. Further limitations concerning the study design and population can also be criticized. However, if we compare the psoriasis and rheumatology patients, we find similar characteristics (e.g., targeted and nontargeted therapies and supplement use) that make the inclusion of dermatology clinic patients a reasonable choice to increase the number of patients. We assessed the SmPC-s of the medicines and found that in 2018–2019, 10 products were used, and 6 were in both indications, whereas in 2022, 11 products were used, and 6 were authorized for psoriasis and arthritis as well.

Another limitation of this study was that we only assessed the pDDIs, and patients were not followed-up or re-evaluated for the occurrence of adverse drug reactions; therefore, we did not collect all the evidence on how many pDDIs would lead to adverse drug reactions.

## 4. Materials and Methods

### 4.1. Study Design

This cross-sectional, observational, prospective study included 185 patients from immunological (rheumatology and dermatology) outpatient clinics receiving targeted therapy at the point of dispensing in one of the satellite pharmacy units of the Central Clinical Pharmacy of the University of Pécs, Hungary. Patients were offered participation in the study if a drug in their therapeutic regimen was a targeted agent, and their age was ≥ 18 years. Data collection was performed in two phases: the first in 2018 and 2019 (n = 100) and the second in 2022 (n = 85). The Institutional Research and Ethics Committee approved the research protocols during both periods (registration numbers: 6964-PTE 2018. and 9007-PTE 2021.). An additional data protection license was applied for and granted in 2022 (KK/1225-2/204). Written informed consent was obtained from all participants.

### 4.2. Data Collection

The project’s academic investigator recruited and trained data collectors (pharmacists, a pharmacy technician specialized in dispensing targeted therapy products, and 4th/5th-year pharmacy students). The research group developed a data collection form based on previous studies [27,28]. Data collected included patient demographics (e.g., age and sex), socioeconomic status, disease, date of diagnosis, disease activity index, prescription drugs, duration of targeted therapy, therapeutic changes in targeted therapy, vaccination status, over-the-counter drugs, and complementary products (e.g., dietary supplements, herbal products, and vitamins). For over-the-counter medications and supplements, only the 2-week period immediately before the interview was assessed. Over-the-counter products authorized as medicines were distinguished from non-medical health products. The latter category included dietary supplements, medical devices, formulas, special foods, teas/species, biocide products, mineral waters, and cosmetics.

The questionnaire was developed based on previous experiences and a literature search. Researchers experienced in pharmacist–patient communication and clinical pharmacy developed the questionnaire, which was pilot-tested among pharmacy students (n = 3) participating in data collection and patients (n = 3) before finalizing the data collection method. No changes were made. Our aim with data collection was to incorporate as many data sources as possible to increase the reliability of the medical information.

Data were collected through structured face-to-face interviews with 37-item (2018–2019) and 33-item (2022) questionnaires before administering targeted therapy. The average duration of patient interviews was 25 min. The medication and supplement use questionnaires were the same, except that we added a COVID-19 section in 2022. To obtain a comprehensive overview of the patients’ medical data, two additional sources of information were utilized: outpatient medical records and the itemized reimbursement reporting interface of the National Health Insurance Fund.

### 4.3. Polypharmacy and Potential Drug–Drug Interactions

Polypharmacy is defined as the concomitant use of ≥5 medications [75,76,77,78].

In our study, similar to other publications, a potential drug–drug interaction (pDDI) is defined as the occurrence of a potentially harmful combination of prescribed drugs in a given patient rather than the occurrence of an actual adverse event in a patient [38]. Vaccines were not included in the polypharmacy and drug–drug interaction screening, as in 2018 and 2022. Most of the databases did not include any information regarding these products, and immunization is usually an acute medical procedure and did not qualify for the 2-week timeframe (see OTC products).

Three different drug interaction software programs were used to assess pDDI: the UpToDate Lexicomp database (Wolters Kluwer Clinical Drug Information), the Medscape drug interaction checker (WebMD LLC.), and Drugs.com databases (Drugsite Limited) (UpToDate Lexicomp; Medscape’s drug interaction checker; Drugs.com). The University of Pécs has licensed access to the UpToDate Lexicomp database [79,80,81].

If a combination (for products containing more than one active ingredient) was not present in the database when queried, each active ingredient was treated as a separate drug.

UpToDate classifies interactions into X (avoid combination), D (consider therapy modification), C (monitor therapy), B (no action required), and A (no known interaction). According to escape, interactions are divided into four categories: contraindicated, serious—use alternative, monitor, and minor. In the case of Drugs.com, interactions are categorized as major, moderate, minor, and unknown. A potential drug–drug interaction was considered severe if it was classified as one of the following categories in one of the databases: as category X or D in UpToDate, contraindicated or serious—use alternative in Medscape, or major in Drugs.com [79,80,81].

The definition of targeted therapy used in this study is any biological or small-molecule disease-modifying agent used in the pharmacotherapy of chronic inflammatory immunology disorders like psoriasis or rheumatoid arthritis (e.g., adalimumab, etanercept, and upadacitinib) [82].

### 4.4. Data Analysis

The descriptive statistics are presented as means and standard deviations (SDs) or percentages. The characteristics of patients in different groups were compared using the chi-square test for categorical variables, and the odds ratio was measured to assess the effect of age, sex, polypharmacy, and supplement use as risk factors for severe pDDIs, potential drug–supplement interactions, and targeted drug interactions. A *p*-value of 0.01 was considered statistically significant. All analyses were performed using SPSS version 26.

## 5. Conclusions

Although methotrexate remains the gold standard for immunology and rheumatology, several new therapeutic approaches are emerging. Additionally, the coronavirus 2019 (COVID-19) pandemic has led to the development of new vaccines. However, screening methods and drug–drug interaction databases are lagging behind these advancements. Therefore, the Summary of Product Characteristics or a single database is insufficient for comprehensive assessment. It is essential to include these therapies along with supplements used by patients and to optimize the vaccination status in the medication review process for patients with chronic inflammation receiving targeted therapies. Adding real-world evidence on drug–drug and drug–supplement interactions to screening databases is essential for developing successful tools for clinical pharmacists. As the range of therapies continues to expand, studies focusing on the potential factors leading to effective outcomes or therapeutic failure are needed. The use of biosimilar or follow-on targeted medicinal products further complicates the situation. Information on the prevalence and risk factors associated with pDDIs in a country-specific or disease-specific context can help implement targeted interventions such as clinical pharmacy services to optimize the long-term therapy of these special patient populations as well as undergraduate or postgraduate educational programs for healthcare professionals.

## Figures and Tables

**Table 1:** Main demographic and therapeutic characteristics of patients included in the survey.

	2018–2019 (N = 100)	2022 (N = 85)	Total (N = 185)	*p*-Value
**Sex (N, %)**				*p* = 0.366
male	43	31	74 (40.0%)	
female	57	54	111 (60.0%)	
**Age groups (N, %)**				*p* = 0.417
<20	1	1	2 (1.1%)	
20–29	3	3	6 (3.2%)	
30–39	7	6	13 (7.0%)	
40–49	19	13	32 (17.3%)	
50–59	33	16	49 (26.5%)	
60–69	23	27	50 (27.0%)	
70–79	13	17	30 (16.2%)	
≥80	1	2	3 (1.6%)	
**Clinic**				*p* < 0.001
Dermatology	31	0	31 (16.8%)	
Rheumatology and immunology	69	85	154 (83.2%)	
**Smoker (N, %)**	25 (25%)	19 (22.4%)	44 (23.8%)	*p* = 0.673
**Medicinal products used (mean ± SD, range)**				
Prescribed medications	6.02 ± 4.28 (0–22)	6.86 ± 4.89 (1–23)	6.41 ± 4.59 (0–23)	
OTC medicines	1.40 ± 1.35 (0–5)	0.99 ± 0.96 (0–4)	1.21 ± 1.22 (0–5)	
Supplementary products	0.52 ± 1.04 (0–6)	2.41 ± 1.42 (0–7)	1.39 ± 1.54 (0–7)	
**Polypharmacy (N, %)**	57 (57.0%)	69 (81.2%)	126 (68.1%)	*p* < 0.001
**Diagnosis (n)**				*p* < 0.001
Rheumatoid arthritis	39	48	87 (47.0%)	
Psoriasis vulgaris	28	0	28 (15.1%)	
Ankylosing spondylitis	21	17	38 (20.5%)	
Psoriatic arthritis	6	14	20 (10.8%)	
Other	6	6	12 (6.5%)	
**Therapeutic change in the targeted therapy (%)**	40%	65.9%		
**Immunization (N, %)**				
Influenza vaccine	28 (28%)	34 (44.7%)	62 (33.5%)	*p* = 0.085
Pneumococcus vaccine	0 (0%)	8 (9.4%)	8 (4.3%)	*p* < 0.001
COVID-19 vaccine	N/A	75 (88.2%)	N/A	-

**Table 2:** The identified non-medical health product and supplement use among patients receiving biological or targeted therapy in 2018–2019 and 2022.

2018–2019	Name	Frequency (n = 164)		2022	Name	Frequency (n = 198)	
1	Vitamin D	33	20.1%	1	Vitamin D	61	30.8%
2	Vitamin D with calcium	17	10.4%	2	Vitamin C	38	19.2%
3	Vitamin C	17	10.4%	3	Calcium	25	12.6%
4	Calcium	12	7.3%	4	Magnesium and vitamin B_6_	11	5.6%
5	Magnesium	10	6.1%	5	Multivitamin	10	5.1%
6	Multivitamin	10	6.1%	6	Magnesium	7	3.5%
7	Magnesium and vitamin B_6_	7	4.3%	7	Nettle	6	3.0%
8	*Matricaria chamomilla*	5	3.1%	8	Fish oil	5	2.5%
9	Fish oil	5	3.1%	9	Vitamin B complex	4	2.0%
10	Vitamin B complex	4	2.4%	10	Milk thistle	3	1.5%
11	Aloe vera	2	1.2%	11	Vitamin D with calcium	3	1.5%
12	Vitamin B_1_	2	1.2%	12	Béres drops	2	1.0%
13	Vitamin B_6_	2	1.2%	13	Collagen	2	1.0%
14	Milk thistle	2	1.2%	14	Iron	2	1.0%
15	*Mentha* sp.	2	1.2%	15	Vitamin B_12_	2	1.0%
16	Lemon balm	2	1.2%	16	Vitamin E	2	1.0%
17	Nettle	2	1.2%	17	Calcium and magnesium	1	0.5%
18	Probiotics	2	1.2%	18	Coenzyme Q_10_	1	0.5%
19	Vitamin A	1	0.6%	19	Cranberry	1	0.5%
20	Béres drops	1	0.6%	20	Curcuma	1	0.5%
21	Cabbage	1	0.6%	21	*Echinacea* sp.	1	0.5%
22	Willowherb	1	0.6%	22	Enteral nutrition	1	0.5%
23	Chlorophyll	1	0.6%	23	Ginger	1	0.5%
24	Bearberry	1	0.6%	24	Green tea	1	0.5%
25	Sea thorn	1	0.6%	25	Iodine	1	0.5%
26	Common yarrow	1	0.6%	26	Laxative species	1	0.5%
27	Rosehip	1	0.6%	27	*Oenothera biennis* oil	1	0.5%
28	Common walnut leaves	1	0.6%	28	Vitamin B_1_	1	0.5%
29	Folic acid	1	0.6%	29	Vitamin B_6_	1	0.5%
30	Garlic	1	0.6%	30	Vitamin D with vitamin C	1	0.5%
31	Ginger	1	0.6%				
32	*Gingko biloba*	1	0.6%				
33	Curcuma	1	0.6%				
34	Iron	1	0.6%				
35	Green tea	1	0.6%				
36	Sage	1	0.6%				
37	*Hypericum perforatum*	1	0.6%				

**Table 3:** Relationship between old age, gender, polypharmacy, and supplement use and the occurrence of severe pDDIs, potential drug–supplement interactions, and biological/targeted pDDIs in all the patients (n = 185).

Outcome (Effect/Dependent Variable)	Measured Parameter (Cause/Independent Variable)	Odd Ratio	95% Confidence Interval	*p*-Value
Severe pDDI	Elderly (>60 years)	1.901	1.022–3.537	0.041
	Gender (female)	3.129	1.674–5.849	<0.001
	Polypharmacy	10.500	5.122–21.532	<0.001
	Dietary supplement use	1.532	0.835–2.810	0.167
Dietary supplement interaction	Elderly (>60 years)	3.173	1.732–5.813	<0.001
	Gender (female)	2.651	1.444–4.865	<0.001
	Polypharmacy	11.518	5.167–25.674	<0.001
	Dietary supplement use	5.175	2.746–9.752	<0.001
Targeted drug interaction	Elderly (>60 years)	2.062	1.121–3.794	0.019
	Gender (female)	3.387	1.823–6.292	<0.001
	Polypharmacy	5.276	2.717–10.284	<0.001
	Dietary supplement use	1.251	0.690–2.266	0.460

**Table 4:** Relationship between old age, gender, polypharmacy, and supplement use and the occurrence of severe pDDIs, potential drug–supplement interactions, and biological/targeted pDDIs in rheumatology and immunology patients (n = 154).

Outcome (Effect/Dependent Variable)	Measured Parameter (Cause/Independent Variable)	Odd Ratio	95% Confidence Interval	*p*-Value
Severe pDDI	Elderly (>60 years)	1419	0.691–2.912	0.339
	Gender (female)	2.777	1.340–5.757	0.005
	Polypharmacy	6.291	2.719–14.554	<0.001
	Dietary supplement use	1.545	0.757–3.156	0.231
Dietary supplement interaction	Elderly (>60 years)	2.467	1.277–4.766	0.007
	Gender (female)	2.267	1.163–4.420	0.015
	Polypharmacy	21.368	6.129–74.502	<0.001
	Dietary supplement use	5.294	2.642–10.607	<0.001
Targeted drug interaction	Elderly (>60 years)	1.642	0.815–3.304	0.163
	Gender (female)	3.035	1.491–6.178	0.002
	Polypharmacy	2.482	1.112–3.674	0.024
	Dietary supplement use	1.310	0.656–2.641	0.444

## Data Availability

The data will be shared upon request to the corresponding author.

## Supplementary Materials

The following supporting information can be downloaded at: https://www.mdpi.com/article/10.3390/ph17070942/s1, Table S1. Selected potential drug-drug interaction with conventional DMARD therapy identified in 2018–2019; Table S2. Selected potential drug-drug interaction with conventional DMARD therapy identified in 2022; Table S3. Selected potential drug-drug interaction with t/bDMARD therapy identified in 2018–2019; Table S4. Selected potential drug-drug interaction with t/bDMARD therapy identified in 2022; Table S5. Selected potential supplement-drug interactions identified in 2018–2019; Table S6. Selected potential drug-drug interaction with t/bDMARD therapy identified in 2022
