# Supplementary material for: Survey of Potential Drug Interactions, Use of Non-Medical Health Products, and Immunization Status among Patients Receiving Targeted Therapies
Source: Pharmaceuticals (Basel). 2024 Jul 14;17(7):942. doi: 10.3390/ph17070942 (PMC11279607; doi:10.3390/ph17070942)
Supplement: Supplementary file 1 [file pharmaceuticals-17-00942-s001.zip › pharmaceuticals-3079672-supplementary.pdf]

Table S1. Selected potential drug-drug interaction with conventional DMARD therapy identified in 2018-2019.

Drug 1 (or therapeutic group)	Drug 2 (or therapeutic group)	Drug interaction
alprazolam	tramadol	CNS Depressants may enhance the CNS depressant effect of Opioid Agonists (UpToDate Lexicomp).
Proton-pump inhibitors (PPIs)	mesalamine	PPIs decrease effects of mesalamine by increasing gastric pH. Applies only to oral form of both agents. Avoid or Use Alternate Drug. Applies only to sustained release dosage form (Medscape drug interaction checker).
Angiotensin-converting enzyme inhibitors (ACEIs)	allopurinol	Angiotensin-Converting Enzyme Inhibitors may enhance the potential for allergic or hypersensitivity reactions to Allopurinol (UpToDate Lexicomp).
Proton-pump inhibitors (PPIs)	methotrexate	Inhibitors of the Proton Pump (PPIs and PCABs) may increase the serum concentration of Methotrexate.<br>The clinical significance of this interaction may be lower with the typically lower antirheumatic methotrexate doses (UpToDate Lexicomp).
Nonsteroidal anti-inflammatory drugs (NSAIDs)	methotrexate	Nonsteroidal Anti-Inflammatory Agents may increase the serum concentration of Methotrexate.<br>The severity of this interaction is likely greater with higher dose methotrexate. Coadministration of NSAIDs and higher doses of methotrexate, such as used those in the treatment of oncologic disorders, should be avoided (UpToDate Lexicomp).
Nonsteroidal anti-inflammatory drugs (NSAIDs)	Angiotensin-converting enzyme inhibitors (ACEIs)	Angiotensin-Converting Enzyme Inhibitors may enhance the adverse/toxic effect of Nonsteroidal Anti-Inflammatory Agents. Specifically, the combination may result in a significant decrease in renal function. Nonsteroidal Anti-Inflammatory Agents may diminish the antihypertensive effect of Angiotensin-Converting Enzyme Inhibitors (UpToDate Lexicomp).
sulfasalazine	methotrexate	Sulfasalazine may enhance the hepatotoxic effect of Methotrexate (UpToDate Lexicomp).
leflunomide	methylprednisolone	Corticosteroids (Systemic) may enhance the immunosuppressive effect of Leflunomide. This interaction applies to systemic corticosteroids at a dose equivalent to more than 2 mg/kg or 20 mg/day of prednisone (for persons over 10 kg) given for more than 2 weeks (UpToDate Lexicomp).
Selective COX-2 inhibitors (coxibs)	Nonsteroidal anti-inflammatory drugs (NSAIDs)	Nonsteroidal Anti-Inflammatory Agents may enhance the adverse/toxic effect of other Nonsteroidal Anti-Inflammatory Agents. Specifically, the risk for gastrointestinal toxicity is increased (UpToDate Lexicomp).
methotrexate	metamizole/dipyrone	Methotrexate may enhance the adverse/toxic effect of Dipyrone. Specifically, the risk for agranulocytosis and pancytopenia may be increased. Dipyrone may enhance the adverse/toxic effect of Methotrexate (UpToDate Lexicomp).

Table S2. Selected potential drug-drug interaction with conventional DMARD therapy identified in 2022.

Drug 1 (or therapeutic group)	Drug 2 (or therapeutic group)	Drug interaction
pregabalin	tramadol	CNS Depressants may enhance the CNS depressant effect of Opioid Agonists (UpToDate Lexicomp).
Proton-pump inhibitors (PPIs)	mesalamine	PPIs decrease effects of mesalamine by increasing gastric pH. Applies only to oral form of both agents. Avoid or Use Alternate Drug. Applies only to sustained release dosage form (Medscape drug interaction checker).
Angiotensin-converting enzyme inhibitors (ACEIs)	allopurinol	Angiotensin-Converting Enzyme Inhibitors may enhance the potential for allergic or hypersensitivity reactions to Allopurinol (UpToDate Lexicomp).
Proton-pump inhibitors (PPIs)	methotrexate	Inhibitors of the Proton Pump (PPIs and PCABs) may increase the serum concentration of Methotrexate.<br>The clinical significance of this interaction may be lower with the typically lower antirheumatic methotrexate doses (UpToDate Lexicomp).
Nonsteroidal anti-inflammatory drugs (NSAIDs)	methotrexate	Nonsteroidal Anti-Inflammatory Agents may increase the serum concentration of Methotrexate.<br>The severity of this interaction is likely greater with higher dose methotrexate. Coadministration of NSAIDs and higher doses of methotrexate, such as used those in the treatment of oncologic disorders, should be avoided (UpToDate Lexicomp).
Nonsteroidal anti-inflammatory drugs (NSAIDs)	Angiotensin-converting enzyme inhibitors (ACEIs)	Angiotensin-Converting Enzyme Inhibitors may enhance the adverse/toxic effect of Nonsteroidal Anti-Inflammatory Agents. Specifically, the combination may result in a significant decrease in renal function. Nonsteroidal Anti-Inflammatory Agents may diminish the antihypertensive effect of Angiotensin-Converting Enzyme Inhibitors (UpToDate Lexicomp).
sulfasalazine	methotrexate	Sulfasalazine may enhance the hepatotoxic effect of Methotrexate (UpToDate Lexicomp).
leflunomide	methylprednisolone	Corticosteroids (Systemic) may enhance the immunosuppressive effect of Leflunomide. This interaction applies to systemic corticosteroids at a dose equivalent to more than 2 mg/kg or 20 mg/day of prednisone (for persons over 10 kg) given for more than 2 weeks (UpToDate Lexicomp).
Selective COX-2 inhibitors (coxibs)	Nonsteroidal anti-inflammatory drugs (NSAIDs)	Nonsteroidal Anti-Inflammatory Agents may enhance the adverse/toxic effect of other Nonsteroidal Anti-Inflammatory Agents. Specifically, the risk for gastrointestinal toxicity is increased (UpToDate Lexicomp).
hydroxychloroquine	Other immunosuppressive agents	Immunosuppressive agents and hydroxychloroquine sulfate both increase immunosuppressive effects; risk of infection (Medscape drug interaction checker).

Table S3. Selected potential drug-drug interaction with t/bDMARD therapy identified in 2018-2019.

Drug 1 (or therapeutic group)	Drug 2 (or therapeutic group)	Drug interaction
adalimumab or other TNF alpha inhibitor monoclonal antibody	alprazolam	Plasma concentrations of drugs that are CYP450 substrates may decrease following the initiation of interleukin (IL) inhibitors, tumor necrosis factor (TNF) blockers, or interferon (IFN) inhibitors in patients with chronic inflammatory diseases. Because the formation of hepatic CYP450 enzymes is down-regulated during infection and chronic inflammation by increased levels of certain cytokines (e.g., interleukins-1, -6, and -10; tumor necrosis factor alpha; interferons), treatment targeting these cytokines may restore or normalize CYP450 enzyme levels resulting in increased metabolism of these drugs (Drugs.com).
adalimumab or other TNF alpha inhibitor monoclonal antibody	amlodipine	Plasma concentrations of drugs that are CYP450 substrates may decrease following the initiation of interleukin (IL) inhibitors, tumor necrosis factor (TNF) blockers, or interferon (IFN) inhibitors in patients with chronic inflammatory diseases. Because the formation of hepatic CYP450 enzymes is down-regulated during infection and chronic inflammation by increased levels of certain cytokines (e.g., interleukins-1, -6, and -10; tumor necrosis factor alpha; interferons), treatment targeting these cytokines may restore or normalize CYP450 enzyme levels resulting in increased metabolism of these drugs (Drugs.com).
adalimumab or other TNF alpha inhibitor monoclonal antibody	chloroquine	The risk of peripheral neuropathy may be increased during concurrent use of two or more agents that are associated with this adverse effect. Patient risk factors include diabetes and age older than 60 years. In some cases, the neuropathy may progress or become irreversible despite discontinuation of the medications (Drugs.com).
adalimumab or other TNF alpha inhibitor monoclonal antibody	leflunomide	Coadministration of leflunomide with other immuno- or myelosuppressive antirheumatic agents may potentiate the risk of infections. Serious infections including sepsis, as well as opportunistic infections like Pneumocystis jiroveci pneumonia, pulmonary and extrapulmonary tuberculosis, and aspergillosis have been reported with the use of leflunomide, particularly in patients on concomitant hematotoxic therapy (Drugs.com).
etanercept	rosuvastatin	The risk of peripheral neuropathy may be increased during concurrent use of two or more agents that are associated with this adverse effect. Patient risk factors include diabetes and age older than 60 years. In some cases, the neuropathy may progress or become irreversible despite discontinuation of the medications (Drugs.com).
adalimumab or other TNF alpha inhibitor monoclonal antibody	methotrexate	The use of tumor necrosis factor (TNF) blockers with other immunosuppressive or myelosuppressive agents may increase the risk of infections. Serious infections and sepsis, including fatalities, have been reported with the use of TNF blockers, particularly in patients on concomitant immunosuppressive therapy (Drugs.com).
adalimumab or other TNF alpha inhibitor monoclonal antibody	methylprednisolone	The use of tumor necrosis factor (TNF) blockers with other immunosuppressive or myelosuppressive agents may increase the risk of infections. Serious infections and sepsis, including fatalities, have been reported with the use of TNF blockers, particularly in patients on concomitant immunosuppressive therapy (Drugs.com).
adalimumab or other TNF alpha inhibitor monoclonal antibody	atorvastatin	Plasma concentrations of drugs that are CYP450 substrates may decrease following the initiation of interleukin (IL) inhibitors, tumor necrosis factor (TNF) blockers, or interferon (IFN) inhibitors in patients with chronic inflammatory diseases. Because the formation of hepatic CYP450 enzymes is down-regulated during infection and chronic inflammation by increased levels of certain cytokines (e.g., interleukins-1, -6, and -10; tumor necrosis

		factor alpha; interferons), treatment targeting these cytokines may restore or normalize CYP450 enzyme levels resulting in increased metabolism of these drugs (Drugs.com).
etanercept	atorvastatin	Plasma concentrations of drugs that are CYP450 substrates may decrease following the initiation of interleukin (IL) inhibitors, tumor necrosis factor (TNF) blockers, or interferon (IFN) inhibitors in patients with chronic inflammatory diseases. Because the formation of hepatic CYP450 enzymes is down-regulated during infection and chronic inflammation by increased levels of certain cytokines (e.g., interleukins-1, -6, and -10; tumor necrosis factor alpha; interferons), treatment targeting these cytokines may restore or normalize CYP450 enzyme levels resulting in increased metabolism of these drugs (Drugs.com).
adalimumab or other TNF alpha inhibitor monoclonal antibody	azathioprine	The use of tumor necrosis factor (TNF) blockers with other immunosuppressive or myelosuppressive agents may increase the risk of infections. Serious infections and sepsis, including fatalities, have been reported with the use of TNF blockers, particularly in patients on concomitant immunosuppressive therapy (Drugs.com).

Table S4. Selected potential drug-drug interaction with t/bDMARD therapy identified in 2022.

**Drug 1 (or therapeutic group)**	**Drug 2 (or therapeutic group)**	**Drug interaction**
adalimumab or other TNF alpha inhibitor monoclonal antibody	alprazolam	Plasma concentrations of drugs that are CYP450 substrates may decrease following the initiation of interleukin (IL) inhibitors, tumor necrosis factor (TNF) blockers, or interferon (IFN) inhibitors in patients with chronic inflammatory diseases. Because the formation of hepatic CYP450 enzymes is down-regulated during infection and chronic inflammation by increased levels of certain cytokines (e.g., interleukins-1, -6, and -10; tumor necrosis factor alpha; interferons), treatment targeting these cytokines may restore or normalize CYP450 enzyme levels resulting in increased metabolism of these drugs (Drugs.com).
adalimumab or other TNF alpha inhibitor monoclonal antibody	amlodipine	Plasma concentrations of drugs that are CYP450 substrates may decrease following the initiation of interleukin (IL) inhibitors, tumor necrosis factor (TNF) blockers, or interferon (IFN) inhibitors in patients with chronic inflammatory diseases. Because the formation of hepatic CYP450 enzymes is down-regulated during infection and chronic inflammation by increased levels of certain cytokines (e.g., interleukins-1, -6, and -10; tumor necrosis factor alpha; interferons), treatment targeting these cytokines may restore or normalize CYP450 enzyme levels resulting in increased metabolism of these drugs (Drugs.com).
adalimumab or other TNF alpha inhibitor monoclonal antibody	atorvastatin	Plasma concentrations of drugs that are CYP450 substrates may decrease following the initiation of interleukin (IL) inhibitors, tumor necrosis factor (TNF) blockers, or interferon (IFN) inhibitors in patients with chronic inflammatory diseases. Because the formation of hepatic CYP450 enzymes is down-regulated during infection and chronic inflammation by increased levels of certain cytokines (e.g., interleukins-1, -6, and -10; tumor necrosis factor alpha; interferons), treatment targeting these cytokines may restore or normalize CYP450 enzyme levels resulting in increased metabolism of these drugs (Drugs.com).
adalimumab or other TNF alpha inhibitor monoclonal antibody	duloxetine/gabapentin	Plasma concentrations of drugs that are CYP450 substrates may decrease following the initiation of interleukin (IL) inhibitors, tumor necrosis factor (TNF) blockers, or interferon (IFN) inhibitors in patients with chronic inflammatory diseases. Because the formation of hepatic CYP450 enzymes is down-regulated during infection and chronic inflammation by increased levels of certain cytokines (e.g., interleukins-1, -6, and -10; tumor necrosis factor alpha; interferons), treatment targeting these cytokines may restore or normalize CYP450 enzyme levels resulting in increased metabolism of these drugs (Gupta et al., 2013; Armanious and Vender, 2021)
Janus kinase (JAK) inhibitors	prevention for cardiovascular disease	JAK inhibitors are effective in the treatment of IMIDs of the skin; however, their safety profile is concerning for an increased risk of major adverse cardiovascular events (MACE), venous thromboembolic events (VTE), serious infections, malignant neoplasm, and death (Yang et al., 2023).
Janus kinase (JAK) inhibitors	methylprednisolone	Coadministration of baricitinib or tofacitinib with other immuno- or myelosuppressive agents may potentiate the risk of infections as well as lymphoma and other malignancies. Serious and sometimes fatal infections due to bacterial, mycobacterial, invasive fungal, viral, or other opportunistic pathogens have been reported in patients receiving baricitinib and tofacitinib, most of whom were taking concomitant immunosuppressants such as methotrexate or corticosteroids (Drugs.com).

adalimumab or other TNF alpha inhibitor monoclonal antibody	leflunomide	Coadministration of leflunomide with other immuno- or myelosuppressive antirheumatic agents may potentiate the risk of infections. Serious infections including sepsis, as well as opportunistic infections like Pneumocystis jiroveci pneumonia, pulmonary and extrapulmonary tuberculosis, and aspergillosis have been reported with the use of leflunomide, particularly in patients on concomitant hematotoxic therapy (Drugs.com).
etanercept	semaglutide and other antidiabetics	Potential etanercept-induced hypoglycemia in diabetic patients (Pfeifer et al., 2017).
secukinumab	CYP450 substrates	Plasma concentrations of drugs that are CYP450 substrates may decrease following the initiation of interleukin (IL) inhibitors, tumor necrosis factor (TNF) blockers, or interferon (IFN) inhibitors in patients with chronic inflammatory diseases. Because the formation of hepatic CYP450 enzymes is down-regulated during infection and chronic inflammation by increased levels of certain cytokines (e.g., interleukins-1, -6, and -10; tumor necrosis factor alpha; interferons), treatment targeting these cytokines may restore or normalize CYP450 enzyme levels resulting in increased metabolism of these drugs (Drugs.com).
ixekizumab	CYP450 substrates	Plasma concentrations of drugs that are CYP450 substrates may decrease following the initiation of interleukin (IL) inhibitors, tumor necrosis factor (TNF) blockers, or interferon (IFN) inhibitors in patients with chronic inflammatory diseases. Because the formation of hepatic CYP450 enzymes is down-regulated during infection and chronic inflammation by increased levels of certain cytokines (e.g., interleukins-1, -6, and -10; tumor necrosis factor alpha; interferons), treatment targeting these cytokines may restore or normalize CYP450 enzyme levels resulting in increased metabolism of these drugs (Drugs.com).

Table S5. Selected potential supplement-drug interactions identified in 2018-2019.

Supplement of phytotherapeutic agent	Drug 2 (or therapeutic group)	Drug interaction
Vitamin D	Magnesium	Increased risk of hypermagnesemia especially in patients with impaired kidney function (Medscape drug interaction checker).
St John's Wort	verapamil	St John's Wort decreases the level or effect of verapamil by affecting hepatic/intestinal enzyme CYP3A4 metabolism (Medscape drug interaction checker).
St John's Wort	atorvastatin	St John's Wort decreases the level or effect of atorvastatin by inducing hepatic/intestinal enzyme CYP3A4 metabolism and reduce absorption by P-glycoprotein (MDR1) efflux transporter (Medscape drug interaction checker).
Vitamin D	Calcium	Generally beneficial combination, in some patient risk of hypercalcemia (Medscape drug interaction checker).
Vitamin D	indapamide	Indapamide may increase effects of vitamin D resulting hypercalcemia (Medscape drug interaction checker).
Omega-3 Fatty Acids	anticoagulant (e.g.: rivaroxaban), antiplatelet drugs (e.g.: clopidogrel)	Omega-3 fatty acids may potentiate the pharmacologic effects of drugs affecting coagulation, increased risk of bleeding (Medscape drug interaction checker).
Vitamin B_{12}	Proton-pump inhibitors (PPIs)	PPIs may inhibit the gastrointestinal absorption of Vitamin B_{12} by reducing the gastric acid secretion (Medscape drug interaction checker).
Vitamin B_{12}	metformin	Metformin may decrease level of Vitamin B_{12} by unspecified interaction mechanism (Medscape drug interaction checker).
Calcium	Beta-blockers (e.g., metoprolol)	Calcium may decrease the oral bioavailability of beta-blockers (Medscape drug interaction checker).
Milk Thistle	Proton-pump inhibitors (PPIs)	Milk Thistle may decrease effects of PPIs by pharmacodynamic antagonism (Medscape drug interaction checker).

Table S6. Selected potential drug-drug interaction with t/bDMARD therapy identified in 2022.

**Supplement of phytotherapeutic agent**	**Drug 2 (or therapeutic group)**	**Drug interaction**
Omega-3 Fatty Acids	Pentoxifylline	Omega-3 fatty acids may potentiate the pharmacologic effects of anticoagulants and other drugs that affect hemostasis (Drugs.com).
Grapefruit Juice	Janus kinase (JAK) inhibitors	Grapefruit Juice as a CYP3A4 inhibitor may increase the serum concentration of JAK inhibitors (Medscape drug interaction checker).
Vitamin D	Magnesium	Increased risk of hypermagnesemia especially in patients with impaired kidney function (Medscape drug interaction checker).
Vitamin D	Calcium	Generally beneficial combination, in some patient risk of hypercalcemia (Medscape drug interaction checker).
Vitamin D	indapamide	Indapamide may increase effects of vitamin D resulting hypercalcemia (Medscape drug interaction checker).
Omega-3 Fatty Acids	anticoagulant (e.g.: rivaroxaban), antiplatelet drugs (e.g.: clopidogrel)	Omega-3 fatty acids may potentiate the pharmacologic effects of anticoagulants and other drugs that affect hemostasis (Medscape drug interaction checker).
Vitamin B_{12}	Proton-pump inhibitors (PPIs)	PPIs may inhibit the gastrointestinal absorption of Vitamin B_{12} by reducing the gastric acid secretion (Medscape drug interaction checker).
Vitamin B_{12}	metformin	Metformin may decrease level of Vitamin B_{12} by unspecified interaction mechanism (Medscape drug interaction checker).
Calcium	Beta-blockers (e.g., metoprolol)	Calcium may decrease the oral bioavailability of beta-blockers (Medscape drug interaction checker).
Milk Thistle	Proton-pump inhibitors (PPIs)	Milk Thistle may decrease effects of PPIs by pharmacodynamic antagonism (Medscape drug interaction checker). (Medscape database only contains other thistles!)
